# 异基因造血干细胞移植治疗Shwachman Diamond综合征伴骨髓增生异常综合征1例

**DOI:** 10.3760/cma.j.issn.0253-2727.2023.01.017

**Published:** 2023-01

**Authors:** 明 周, 远文 蒋, 建军 陈, 超 吴, 彬镔 邹, 钊 陈, 林 李, 平 雷, 广花 刘, 艳艳 田, 满丽 朱, 灿 刘

**Affiliations:** 湖南省人民医院（湖南师范大学附属第一医院）血液科，长沙 410000 Department of Hematology, Hunan Provincial People's Hospital (the First Affiliated Hospital of Hunan Normal University), Changsha 410000, Chian

患者，女，27岁，主因“反复血细胞减少15年，发热、牙龈肿痛1周”于2020年6月以“全血细胞减少待查”收住院。血常规示WBC 1.44×10^9^/L、RBC 2.22×10^12^/L、HGB 75 g/L、红细胞平均体积（MCV）99.7 fl、平均红细胞血红蛋白浓度（MCHC）356 g/L、平均红细胞血红蛋白含量（MCH）35.6 pg、PLT 13×10^9^/L。入院查体：贫血貌。全身浅表淋巴结未触及。左侧牙龈可见肿胀、出血。心肺腹未见明显异常。血糖（空腹）6.57 mmol/L，乳酸脱氢酶346.45 U/L，血钾3.34 mmol/L，钠136 mmol/L，氯98 mmol/L，尿酸478 µmol/L；余肝肾功能、心肌酶谱指标正常；纤维蛋白原4.14 g/L；血清铁蛋白（SF）1691.47 µg/L，C反应蛋白89.9 mg/L，降钙素原、免疫球蛋白、肿瘤标志物、叶酸、维生素B12、淋巴细胞亚群、尿便常规均正常；类风湿因子、抗核抗体、狼疮全套、血及尿免疫固定电泳、EB病毒、巨细胞病毒、呼吸道腺病毒、甲乙流感病毒均阴性。骨髓象：骨髓增生明显活跃，原始中性粒细胞占6.5％，粒、红系可见病态造血。骨髓活检：骨髓增生活跃，可见不成熟前体细胞异常定位现象。免疫分型：可见幼稚细胞分布，约占有核细胞5.14％，表达CD34^+^、CD117^+^、CD13p^+^、CD33^+^、HLA-DRdim^+^，不表达CD11b、CD4、CD2、CD5、CD3、CD15、CD8、CD7、CD64、CD33、CD16、CD1a、CD14、CD20、CD38、CD138、CD10、CD19、CD22、CD56，部分粒细胞考虑表型异常。染色体核型：41～48，XX，−5，−6，del（7）（q32），+8，−10，−21，−22，+2～5mar，inc［cp7］/46，XX[3]。心脏彩超：三尖瓣轻度返流，左心功能测值正常；头胸腹增强CT未见异常。患者父母非近亲结婚，家族史及遗传史无异常。

基因检测：患者TP53基因检测到移码突变c.482_483insG（p.Ile162HisfsTer19）和剪切位点突变c.560-1G>A。SBDS基因检测到杂合的剪切位点突变c.258+2T>G（p.Cys84fsTer3）和杂合的缺失插入突变c.183_184delinsCT（p.Lys62Ter）。经家系验证，患者突变c.258+2T>G（p.Cys84fsTer3）遗传自其母亲，突变c.183_184delinsCT（p.Lys62Ter）遗传自其父亲，其父母均只携带其中1个杂合突变，为正常表型。

临床诊断：①Shwachman Diamond综合征；②骨髓增生异常综合征（EB-1）。予地西他滨25 mg·m^−2^·d^−1^×5 d去甲基化治疗2疗程，疗效评估未缓解。患者与其胞弟HLA 10/10相合，且经全外显子测序检查确证无上述突变。2020年11月行异基因造血干细胞移植，回输供者单个核细胞9.97×10^8^/kg，CD34^+^细胞6.93×10^6^/kg。移植后11 d血小板重建，13 d粒细胞重建。移植后21 d短串联重复序列（STR）骨髓嵌合状态检测：供者细胞占99.91％。骨髓SBDS基因外显子2及附近区域未检测到变异；TP53基因外显子5上未检测到移码突变，外显子6未检测到剪切位点突变。移植后2个月复查骨髓完全缓解（CR），微小残留病（MRD）阴性，SBDS基因、TP53基因均阴性。移植后3个月骨髓CR，MRD 0.104％，STR示供者细胞占99.91％（完全嵌合），予快速减停免疫抑制剂，移植后3.5个月复查骨髓CR、MRD 0.272％，于2021年2月20日予地西他滨15 mg/d×5 d治疗，2月26日回输供者淋巴细胞1.2×10^8^/kg。移植后4个月复查骨髓CR，MRD 1.454％，供者细胞占82.19％（混合嵌合状态），SBDS基因、TP53基因阳性。2021年3月23日予维奈克拉+阿扎胞苷化疗，随访至2021年4月，患者因疾病进展死亡。

